# Predictors of vaginal delivery following balloon catheter for labor induction in women with one previous cesarean

**DOI:** 10.1186/s12884-023-05734-y

**Published:** 2023-06-05

**Authors:** Kaidong Ma, Ming Yang, Xiaoling Feng, Liyuan Liu, Liangliang Li, Yunxiu Li

**Affiliations:** 1 Obstetrics department, Shenzhen Longhua District Central Hospital, Shenzhen, China; 2 Obstetrics department, The First Dongguan Affiliated Hospital of Guangdong Medical University, Dongguan, China

**Keywords:** Balloon catheter, Cervical ripening, Induced labor, Cesarean section, Vaginal birth after cesarean

## Abstract

**Background:**

The aim of this study was to estimate predictors for vaginal birth following balloon catheter induction of labor (IOL) in women with one previous cesarean section (CS) and an unfavorable cervix.

**Methods:**

This 4-year retrospective cohort study was conducted in Longhua District Central Hospital in Shenzhen China, between January 2015 and December 2018. Patients with one previous CS and a current singleton-term pregnancy who underwent balloon catheter cervical ripening and IOL were enrolled. Univariate analysis was used to identify predictive factors associated with vaginal birth after cesarean section (VBAC). Binary logistic regression was further used to identify which factors were independently associated with the outcome measure. The primary outcome was VBAC, which was a successful trial of labor after cesarean delivery (TOLAC) following IOL.

**Results:**

A total of 69.57% (208/299) of the women who planned for IOL had VBAC. In the final binary logistic regression equation, lower fetal weight (< 4000 g) (odds ratio [OR]5.26; 95% confidence interval [CI] 2.09,13.27), lower body mass index (BMI,<30 kg/m^2^) (OR 2.27; CI 1.21, 4.26), Bishop score after cervical ripening > 6 (OR 1.94; CI 1.37, 2.76) remained independently associated with an increased chance of VBAC.

**Conclusions:**

The influencing factors of VBAC following IOL were fetal weight, BMI, and Bishop score after cervical ripening. Adequate individualized management and assessment of the IOL may help improve the VBAC rate.

## Introduction

Over the past 20 years, the global CS rate has significantly increased from 12.1 to 21.1% (2000–2015) [1, 2]. According to the World Health Organization survey in 2008, the CS rate in China was reported at 46.2%, placing China among the countries with the highest CS rates in the world [3, 4]. With the installment of the two-child policy in 2016 and the adjustment to the three-child policy in 2021, TOLAC has been increasingly requested by women with prior CS in China [5]. Current evidence suggests that women who undergo repeated CS have a significantly higher risk of maternal and perinatal morbidity than those with VBAC. Major complications associated with TOLAC include hysterectomy and uterine rupture, but successful TOLAC is also associated with decreased maternal morbidity and decreased risk of complications in subsequent pregnancies [6–9].

IOL is a common intervention method in the obstetric management of high-risk pregnancies. Previous studies have shown that 60-80% of women with a previous CS will have a vaginal delivery if there is an opportunity to try labor, even if labor is induced [10–14]. IOL in women with previous CS is generally accepted by various national guidelines but is also associated with an increased risk (around 1%) of uterine rupture [6, 7, 15]. Moreover, an unfavorable cervix is frequently observed at the onset of induction, which increases the risk of CS [16]. Cervical ripening can be obtained by pharmacological or mechanical pharmacological methods [17–19]. The balloon catheter is a mechanical method reported to be associated with less hyperstimulation of the uterus and fewer pathological fetal heart rate abnormalities than prostaglandins [20, 21], which can increase the risk of uterine rupture in women with previous CS [14, 22, 23]. In women without previous CS, balloon catheters and vaginal prostaglandins have comparable CS rates and maternal safety profiles, but balloon catheters lead to fewer adverse perinatal events [18]. Also, guidelines now discourage the use of prostaglandins but suggest using the balloon catheter for cervical ripening in women with previous CS [6, 7].

Although several predictive models have been developed to predict the likelihood of success in TOLAC [24, 25], there is little information on specific factors that may predict IOL in women with previous CS [11, 26]. These factors may assist clinicians in selecting and consulting candidates for IOL in women with previous CS.

Our study aimed to assess the most relevant features of vaginal delivery following balloon catheter IOL in women with previous CS and unfavorable cervix to facilitate the choice of delivery modes for pregnant women.

## Materials and methods

This study has been carried out in accordance with the Declaration of Helsinki (2000) of the World Medical Association and approved by the review boards of Shenzhen Longhua District Central Hospital (IRB2020-135-01) [AF/SQ-02/01.1].

### Study population

This retrospective cohort study was conducted in Longhua District Central Hospital in Shenzhen China between January 2015 and December 2018. Inclusion criteria were as follows: pregnant women with one previous CS and a current cephalic presentation, singleton term pregnancy (between 37 and 41 weeks’gestational age) requiring IOL, with an unfavorable cervix (Bishop score before cervical ripening ≤ 5) [19] and scheduled for IOL. Patients with any of the following were excluded from the study: premature rupture of membranes, preterm labour (gestational age < 37 weeks), two or more CSs, contradictions for a vaginal birth, history of other uterine incisions such as myomectomy, and incomplete medical records.

### Treatment principles and methods

The principles and methods followed by the hospital in carrying out IOL with previous CS included: (1) a doctor (with the title of deputy director or above) evaluated the pre-induction assessment); (2) teams were trained to perform emergency CS in case of fetal distress, threatened uterine rupture, or uterine rupture; (3) patients were informed about the advantages and disadvantages of IOL and signed informed consent; (4) IOL method: the catheter was placed transcervically, followed by fetal heart rate tracing. A single balloon (Foley) catheter (18 F, n = 180) and a double-balloon (Cook) catheter (n = 119) were used. The single-balloon catheters and double-balloon catheters were filled with 60 ml of sterile saline. The cervix was examined after balloon catheter removal 12 h after placement or after the spontaneous expulsion of the balloon catheter into the vagina. If premature rupture of membranes occurred during or after the balloon placement, the balloon catheter was removed. Bishop score before cervical ripening was determined at the last exam before the balloon catheter was placed; Bishop score after cervical ripening was determined at the time of balloon catheter removal or spontaneous expulsion. Even if the Bishop score after cervical ripening remained < 6 after 12 h, induction was pursued by oxytocin perfusion. If uterine activity was insufficient (< 2 times contractions per 10 min), oxytocin was given intravenously until contractions occurred 3 to 4 times in 10 min or sufficient progression was observed. The rate of oxytocin infusion should be ≤ 20 mU/min. (5) Fetal distress was defined as Category III tracings of electronic fetal heart rate monitoring or persistent Category II tracings [27]. Failed induction was defined as nonprogression to the active phase at least 12 to 18 h of oxytocin administered after membrane rupture [28]. The arrest of the active phase was defined as starting around 6 centimeters of dilation, absence of cervical change > 4 h in the presence of adequate contractions or 6 h with inadequate contractions [29]. An abnormal second stage of labor was defined as no progress in descent or rotation for 2 h or more in multiparous women without epidural and 3 h or more for multiparous women with epidural analgesia [30]. Placental abruption was defined as the partial or complete placenta detachment from the underlying myometrium before the expected delivery time and was mainly diagnosed based on clinical grounds in a patient experiencing a new onset of antepartum hemorrhage and painful uterine contractions or uterine tenderness, fetal distress or death, and blood clots behind the placenta [31]. Postpartum hemorrhage (PPH) was defined as the total blood loss ≥ 1000 ml within 24 h after the delivery process (including intrapartum loss), regardless of the route of delivery [32].

### Data collection

Demographic characteristics, obstetric characteristics, mode of delivery, and maternal and fetal complications were collected from the computerized medical system. Data collected at baseline included age, education level, BMI, number of pregnancies, history of vaginal delivery, gestational weeks, indications of previous CS, indication for IOL, pregnancy complications and overall complications, fetal weight, and mode of delivery. In addition, data on delivery were collected, including Bishop score before cervical ripening, Bishop score after cervical ripening, oxytocin use, artificial rupture of membranes, etc. The primary outcome was VBAC, which was successful TOLAC following IOL. The secondary outcome was the comparable effect of single balloons versus double balloons in promoting cervical maturation.

### Statistical analysis

SPSS 20.0 software was used for data analysis. The measurement data were compared by paired t-test and variance analysis, and data were expressed as‾x ± s. Comparison of counting data was evaluated by χ^2^ test or Fisher exact probability method, expressed as a percentage (%). The factors related to the successful TOLAC following IOL were analyzed by binary logistic regression. P < 0.05 represented statistically significant difference.

## Results

### Rate of vaginal delivery following IOL

A total of 20,031 pregnant women were analyzed; 2853 pregnant women had CS and the CS rate was 14.24% (2853/20,031). There were 3,636 pregnant women with one or more CS, accounting for 18.16% (3,636/20,031) of the total deliveries. Among the 3636 women, there were 1770 cases of vaginal delivery, and the vaginal delivery rate was 48.7% (1770/3636).

A total of 299 pregnant women with one previous CS and a current singleton-term pregnancy underwent the balloon catheter cervical ripening and IOL. Patients were between 20 and 40 years old, with an average of 29.84 ± 3.62 years old, 37–41 weeks pregnant, with an average of 40.00 ± 0.85 weeks. Bishop score before cervical ripening was 1–5, with an average of 3.55 ± 0.87 scores. Bishop score improvement after cervical ripening was between 0 and 7 points, with an average of 2.78 ± 1.20. There were 208 cases of vaginal delivery following IOL (success of TOLAC following IOL; VBAC group), including 21 (10.10%) cases of forceps delivery in the VBAC group, and the vaginal delivery rate following IOL was 69.57%. There were 91 cases (30.43%) of CS (failure of TOLAC following IOL; CS group). The reasons for CS were failed induction (33, 36.26%), followed by fetal distress (20, 21.98%), abnormal stage of labour (arrest of active phase or abnormal second stage of labor) (18, 20.0%), chorioamnionitis (3, 3.30%), threatened uterine rupture (3, 3.30%), placental abruption (3, 3.30%), and others (11, 12.08%).

### Complications caused by IOL by balloons catheter

The incidence of PPH in pregnant women who underwent balloon induced-labor with previous CS was 2.01% (6/299), the incidence of blood transfusion was 2.34% (7/299), the incidence of maternal infection was 3.34% (10/299), and the incidence of neonatal asphyxia was 0.33% (1/299). No serious complications, such as uterine rupture, hysterectomy, or maternal and perinatal death, were observed.

### Women’s characteristics and single-factor analysis of factors affecting the successful TOLAC following IOL

The demographic and clinical characteristics of women and univariate relationships between successful TOLAC following IOL are shown in Table 1. There were significant differences in BMI, vaginal delivery history, fetal weight, and Bishop score after cervical ripening between the VBAC group and CS group (all P < 0.05, Table 1).

**Table 1:** Women’s characteristics and Single-factor analysis of factors affecting the successful vaginal delivery following IOL

Variable	Success of TOLAC following IOL (VBAC group) (n = 208)	Failure of TOLAC following IOL (CS group) (n = 91)	χ^2^	P
**Age (years)**				
<35	191	79	1.346	0.178
≥ 35	17	12		
**Education level (year)**				
≤9	98	51	1.418	0.156
>9	110	40		
**BMI (kg/m2)**				
<30	179	69	2.161	0.031
≥30	29	22		
**Pregnancy times (Times)**				
2	70	31	0.310	0.857
3	84	34		
≥4	54	26		
**Times of an artificial abortion operation**				
<3	201	84	1.627	0.104
≥3	7	7		
**History of vaginal delivery**				
Have	19	2	2.156	0.031
No	189	89		
**Gestational week (weeks)**				
<40	43	27	1.688	0.091
≥40	165	64		
**Indications of previous CS**				
Failed trial of labor	15	2	5.899	0.117
Fetal distress	27	9		
Abnormal fetal position	28	8		
Other	138	72		
**Indication for IOL**				
Postdates	155	68	6.702	0.153
Diabetes	24	17		
Hypertension	9	2		
Oligohydramnios	7	3		
Other	13	1		
**Pregnancy complications and postoperative complications**				
Have	56	32	0.905	0.366
No	132	59		
**Fetal weight (kg)**				
<4	200	72	4.720	0.000
≥4	8	19		
**Bishop score before cervical ripening (score)**				
≤3	92	46	1.007	0.314
>3	116	45		
**Bishop score after cervical ripening (score)**				
≤6	136	83	4.634	0.000
>6	72	8		
**Duration of oxytocin use (hours)**				
≤12	154	72	0.940	0.347
>12	54	19		
**Obstetrical management**				
None	54	19	3.092	0.213
Oxytocin	76	28		
Artificial membrane rupture	78	44		
**Induced labor balloon category**				
Single- balloon	128	52	0.713	0.476
Double -balloon	80	39		

### Multifactorial logistic regression analysis of factors affecting the successful TOLAC following IOL

The statistically significant variables in the single-factor analysis were included in binary logistic regression analysis for further screening. As shown in Table 2, in the final binary logistic regression equation, Bishop score after cervical ripening > 6 score (OR 1.94; CI 1.37, 2.76), lower BMI (< 30 kg/m2) (OR 2.27; CI 1.21, 4.26), lower fetal weight (< 4000 g) (OR 5.26; CI 2.09, 13.27) remained independently associated with an increased chance of a successful TOLAC following IOL.

**Table 2:** Multifactorial logistic regression analysis factors affecting the successful vaginal delivery following IOL

Factor		Reference	B	S.E.	P	OR	95% CI
**Intercept**			-5.482	1.194	0.000	0.004	---
**History of vaginal delivery**	Have	No	1.413	0.793	0.075	4.109	(0.869,19.429)
**Fetal weight (kg)**	< 4.0	≥ 4.0	1.661	0.472	0.000	5.264	(2.089,13.266)
**BMI (kg / m2)**	< 30	≥ 30	0.818	0.322	0.011	2.265	(1.205,4.258)
**Bishop score after cervical ripening** **(score)**	> 6	≤ 6	0.664	0.180	0.000	1.942	(1.366,2.761)

### Complications of efficacy between the single-balloon catheters and double–balloon catheters

A total of 180 women with one previous CS used single-balloon catheter, and 119 used double-balloon catheter. There were no significant differences in Bishop score before cervical ripening (3.59 ± 0.84 vs. 3.42 ± 0.83), Bishop score after cervical ripening (6.31 ± 0.84 vs. 6.34 ± 0.80), Bishop score increment (2.72 ± 1.27 VS 2.92 ± 1.09), and vaginal delivery rate following IOL (71.1% vs. 67.2%) between single-balloon catheters and double-balloon catheters (all P > 0.05) in Table 3. Both balloon catheters have similar levels of efficacy at cervical ripening.

**Table 3:** Complications of efficacy between the single-balloon catheters and double-balloon catheters

Variable	Single-balloon catheters (n = 180)	Double-balloon catheters (n = 119)	χ^2^/t	P
Bishop score before cervical ripening (score)	3.59 ± 0.84	3.42 ± 0.83	1.770	0.078
Bishop score after cervical ripening (score)	6.31 ± 0.84	6.34 ± 0.80	0.293	0.770
Bishop score increment (score)	2.72 ± 1.27	2.92 ± 1.09	1.466	0.144
vaginal delivery rate following induced labor [n (%)]	128(71.1)	80(67.2)	0.713	0.476

## Discussion

The induction rate for women attempting a vaginal delivery after a previous CS is 18–27% [33]. For women with CS requiring IOL for subsequent pregnancy, current guidelines recommend using balloon catheters to promote cervical maturation [6, 7]. Balloon catheters have been proven effective in women with a previous CS, with vaginal delivery rates of 50-64% [14, 34–37]. In this study, the success rate of vaginal delivery induced by a balloon was about 70%, higher than reported in the literature [14, 34–37]. The high success rate of TOLAC in our research may be related to doctor’s experience (i.e., obstetric department director), who was personally very supportive of vaginal birth, trained the team well, and promoted the use of midwifery techniques, which resulted in the absence of complications in most of the induced labor pregnant women [38]. In their study, Dodd et al. reported that 68% of pregnant women with previous CS were willing to accept IOL [39], as it could help avoid unnecessary repeated CS and improve the success rate of vaginal delivery [40].

### Conflict

ing data exist concerning the safety of IOL in women with previous CS [41–44]. In this study, we did not observe serious complications, such as uterine rupture, hysterectomy, or maternal and perinatal death. Likewise, Wu et al. reported that labor induction could not increase the incidence of maternal and infant complications with re-pregnancy after CS [45].

The biggest impact of failed TOLAC following IOL is emergency CS. Therefore, the ability to predict a woman’s successful TOLAC has an important role for women who need IOL. Previous studies clarified that a previous vaginal birth strongly predicts a successful TOLAC [11, 14, 45, 46]. In this study, single-factor analysis revealed that the success of TOLAC following IOL depended on the history of vaginal delivery. However, in the multiple-factor analysis, the favorable factors for the success of TOLAC were not included the history of vaginal delivery. The root causes include the following two points: first, due to the influence of China’s fertility policy, there are very few women with a history of vaginal delivery and a history of CS. Second, this is a retrospective analysis, and most participants have vague descriptions of whether a vaginal trial was performed during previous CS, so it is impossible to analyze the impact of previous vaginal trial history on VBAC.

Bishop score is an important method that can predict successful IOL and an important factor in predicting the success rate of vaginal delivery [24, 46, 47]. In this study, Bishop score before cervical ripening did not affect the success of TOLAC following IOL. However, if Bishop score after cervical ripening was > 6, the success rate of the delivery was higher (OR 1.94; CI 1.37, 2.76), which suggested that, upon cervical ripening by the balloon catheters, the role of these influencing factors in IOL was altered by a marked improvement in the cervical Bishop score [48]. In this study, the effectiveness of single balloon and double balloon for cervical maturation was compared, and the cervical score of the single balloon group before and after cervical maturation increased by 2.72 ± 1.27, and the cervical score of the double balloon group increased by 2.92 ± 1.09, and there was no significant difference between the two groups. Both single-balloon and double-balloon could effectively promote cervical ripening, which was consistent with De Los et al. [49].

Previous studies have demonstrated that patients undergoing TOLAC with a macrosomic fetus are less likely to achieve VBAC than patients with a nonmacrosomic fetus, with success rates ranging from 38 to 68% [50–53]. On the contrary, a recent study has demonstrated that women attempting TOLAC with a macrosomic neonate are not at increased risk for failed TOLAC and operative and uterine rupture [54]. Recent American College of Obstetrics and Gynecology guidelines do not consider macrosomia as a contraindication to TOLAC [6]. Our results indicated that lower fetal weight (< 4000 g) (OR 5.26; CI 2.09, 13.27) is independently associated with an increased chance of a successful TOLAC following IOL. As was also concluded by Kugelman et al., the birth weight of newborns after TOLAC was one of the factors that were significantly associated with a successful IOL [55].

BMI is another predictor incorporated into our prediction. Lower BMI (< 30 kg/m2) (OR 2.26; CI 1.2, 4.26) was a factor associated with the likelihood of achieving a successful trial of labor. Similarly, previous studies have identified that low maternal BMI was significantly associated with a higher chance of successful TOLAC. Maternal obesity (BMI > 30 kg/m2) is associated with a more difficult IOL process and an increased risk of failed IOL and CS [50, 56–58].

Moreover, studies suggested that women over 40 weeks of gestation were more likely to have a failed TOLAC [59, 60]. Palatnik et al. noted that labor induction at 39 gestational weeks might increase the chances of VBAC. Our analysis revealed that a gestational age of 37–40 weeks was not associated with the success of TOLAC following IOL. Similarly, Ram et al. found that the success of TOLAC was not affected in women over 40 weeks of gestation [61].

Many studies have suggested that primiparity, high BMI, and unfavorable Bishop scores are associated with failed induction in non-previous CS women [28, 62, 63]. On the contrary, Daykan et al. [64] found that the high Bishop score at admission was not associated with cervical ripening in non-previous CS women. A cohort study also showed that a favorable Bishop score after cervical ripening is associated with a decreased rate of CS in non-previous CS women undergoing IOL [19]. This study found that the successful TOLAC after IOL was associated with approximately the same factors related to labor induction without a history of CS. The next step can be to do a prospective study to compare the influencing factors of labor induction success with and without CS.

The critical strength of this study is that all labor inductions made with balloon catheters were filled with 60 ml of sterile saline, and all patients followed and were treated the same way. This reduces prejudices about different management approaches. Another strength of this study is the larger sample size (299 patients were analyzed), which is higher than that reported in previous studies in China. In addition, the success rate of vaginal delivery was higher than that was reported in the literature. The main limitation of this study is its retrospective nature. Also, most participants had vague descriptions of whether the vaginal trial was performed at the time of the previous CS, so it was not possible to analyze the effect of the previous vaginal trial history on VBAC.

## Conclusion

The success rate of TOLAC (69.5%), established following balloon catheter IOL in women with one previous CS and unfavorable cervix, is very high, which implies that IOL is accepted and is an important strategy that may decrease the CS rate in China. The influencing factors of VBAC following IOL are fetal weight, BMI, and Bishop score after cervical ripening. The factors predicting the success of TOLAC generated in the study could be a potential tool for more directed IOL counseling for women with a previous CS. Further prospective validation studies with larger sample sizes and in the general population should be undertaken to confirm efficacy before pervasive application among Chinese women and to estimate maternal and neonatal adverse events of TOLAC after IOL.

## Data Availability

The datasets used and/or analyzed during the current study are available from the corresponding author upon reasonable request.
